# An update on phloem transport: a simple bulk flow under complex regulation

**DOI:** 10.12688/f1000research.12577.1

**Published:** 2017-12-06

**Authors:** Johannes Liesche, John Patrick

**Affiliations:** 1 Biomass Energy Center for Arid and Semi-arid lands, Northwest A&F University, Yangling, China; 2 College of Life Science, Northwest A&F University, Yangling , China; 3 Department of Biological Sciences, School of Environmental and Life Sciences, The University of Newcastle, Callaghan, Australia

**Keywords:** Phloem transport, signalling, plants, water

## Abstract

The phloem plays a central role in transporting resources and signalling molecules from fully expanded leaves to provide precursors for, and to direct development of, heterotrophic organs located throughout the plant body. We review recent advances in understanding mechanisms regulating loading and unloading of resources into, and from, the phloem network; highlight unresolved questions regarding the physiological significance of the vast array of proteins and RNAs found in phloem saps; and evaluate proposed structure/function relationships considered to account for bulk flow of sap, sustained at high rates and over long distances, through the transport phloem.

## Introduction

Successful functioning of land plants depends upon specialized transport systems. With some exceptions such as remobilized reduced carbon in winter-deciduous trees during the winter-spring transition, xylem conducts water and essential mineral elements from roots to transpiring leaves. Phloem transports water, mineral elements, amino nitrogen compounds, and sugars (resources), together with signalling molecules, from fully expanded leaves (sources) to meet the nutrient requirements of heterotrophic growth or storage organs (sinks) and to direct their development, respectively. Thus, phloem transport is at the heart of plant growth and development and, as such, is a primary factor determining crop yield potential. (For recent reviews, see Braun *et al*. ^1^, Ham and Lucas ^2^, and Yadav *et al*. ^3^.)

Phloem transport occurs as a pressure-driven bulk flow through a longitudinally arrayed subset of transport-specialized cells termed sieve elements (SEs). During their development, SEs undergo partial autophagy that leaves a parietal enucleate cytoplasm enclosed by a plasma membrane (PM). (For more details of recent progress in understanding phloem development, see reviews by Rodriguez-Villalon ^4^ and de Rybel *et al*. ^5^.) In contrast to SEs, their adjoining companion cells (CCs) contain a dense cytoplasm and are connected to their corresponding SE through an extensive network of plasmodesmata (PD) to form a metabolic and genetic unit, the sieve element-companion cell complex (SECCC). Loading SECCCs in the collection phloem of source leaves generates an osmotically derived hydrostatic pressure potential that drives bulk flow through the SE arrays of the transport phloem to reach the release phloem where resources and signals are unloaded to enter pathways leading to plant growth or storage ^6, 7^.

In this commentary, we review new insights into key aspects of phloem transport and highlight unresolved questions that need to be addressed to gain a fuller understanding of phloem transport.

## Phloem loading

Sugar uptake into the phloem is a principal contributor to establishing source-sink hydrostatic pressure differentials driving phloem transport ^6, 7^. Different phloem loading types have been identified, and abundant insight into phloem loading mechanisms has become available. However, two key aspects remain largely unexplored: the ecology and regulation of phloem loading.

### Three major types of phloem loading

**Figure 1.**
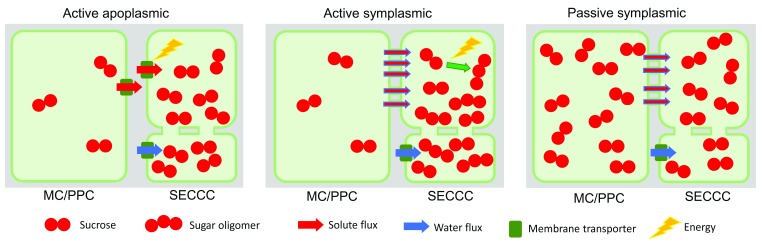
Major phloem loading types. In active apoplasmic loading, the sieve element-companion cell complex (SECCC) is symplasmically isolated. Sucrose produced in the mesophyll cells (MCs) diffuses into phloem parenchyma cells (PPCs), where it is released into the apoplasm by efflux carriers (SWEETs) before being taken up into the SECCC by plasma membrane-localized sucrose transporters. In active symplasmic loading, sucrose can diffuse or convect into the companion cell (CC) through the abundant plasmodesmata (PD). Sucrose in the CC is converted to sugar oligomers, which are hindered from diffusing back into the phloem parenchyma but instead enter the SE through the larger PD at the SE-CC interface. In passive symplasmic loading, sucrose can diffuse or convect along the whole phloem loading pathway from mesophyll cells to SEs following the sugar concentration gradient. Aquaporins facilitate the osmotic uptake of water from the phloem apoplasm into SEs in all loading types. In symplasmic loaders, water also enters the SECCC through PD. SWEET, sugar will eventually be exported transporters.

The three main types of phloem loading currently recognized are (i) active apoplasmic, (ii) active symplasmic, and (iii) passive symplasmic ( Figure 1). The mechanism of active apoplasmic loading ( Figure 1) is well understood with proton-coupled sucrose transporters (SUTs), also referred to as SUCs, concentrating sucrose into SECCCs ^8– 10^ released from surrounding cells by sucrose uniporters, termed sugars will eventually be exported transporters (SWEETs) ^11, 12^. In contrast, the function of the two symplasmic loading types has been established only recently.

A remaining major question for active symplasmic loading is how PD enable steric filtering to allow sucrose movement into CCs while preventing the synthesized sugar oligomers from leaking back to the mesophyll cells ( Figure 1). No method exists to resolve PD canal sub-structure to the degree required to answer this question, and current insights rest solely on mathematical modelling. For instance, Liesche and Schulz ^13^ showed that a PD structure that is highly restrictive to solute movement, with cytosolic channels of 0.65 nm in diameter compared with diameters of 2.5 nm in PD between leaf mesophyll cells ^14^, could enable steric sugar filtering while accommodating observed phloem loading rates. However, this extreme PD anatomy might not be necessary as differences in intracellular sugar concentrations along the loading pathway from mesophyll to phloem could be sufficient to drive a combination of diffusion and mass flow of molecules (convective flow) into SECCCs. As a consequence, diffusion of sugar oligomers out of the SECCC, against the direction of the convective flow, would be limited ^15, 16^.

New evidence regarding passive symplasmic loading ( Figure 1) was provided by the finding that sucrose export from poplar leaves was not compromised by blocking apoplasmic loading of sucrose ^17^. This was achieved through expressing a cell wall invertase to cleave sucrose into hexoses, thereby making the sugar unavailable for uptake by SUTs. Furthermore, microfluidic experiments showed that passive phloem loading is feasible with convective flow through PD to the SECCCs ^18^. However, it is unclear whether this concept is universally applicable across all species exhibiting features of passive symplasmic loading. For example, in several designated passive loaders, higher sugar concentrations were detected in phloem compared with mesophyll cells ^19, 20^. In addition to methodological controversies surrounding measurements of intracellular sugar concentrations, a switch between loading types or even their concomitant operation could explain the conflicting conclusions ^21^.

### Parallel and sequential operation of different phloem loading types

Coexistence of phloem loading types has become apparent for many plant species ^22^. For instance, two anatomical types of CCs have been detected in minor veins in 35% of 320 Asteridae species exhibiting PD characteristics consistent with active symplasmic or apoplasmic loading ^23^ as well as other species of various growth forms ^24, 25^. Moreover, switching from active symplasmic to apoplasmic phloem loading, putatively by modifying PD conductance in the same CC, has been described for virus-infected melon plants ^26^. A switch between loading types in the same CC is thought to be impossible without adaptation of the relevant PD ^21, 27^. Consequently, elucidating this potential mechanism of switching between loading types is a major task for future research on phloem loading.

In addition to switching between loading types, concomitant operation of passive and active loading by the same CC has been proposed ^20^ and shown to be theoretically possible though attenuated by a low efficiency ^21^. Passive loading could function as a fallback mechanism as demonstrated by continued growth, at a reduced rate, of plants in which active loading is blocked through introduction of mutations that render either the relevant SUT or enzymes involved in sucrose oligomerization non-functional ^22, 28, 29^.

### Ecology of phloem loading types

No satisfactory answer has been provided to account for why there are different phloem loading types. Slewinski *et al*. ^22^ theorized that some plants load certain molecules symplasmically in addition to those transported across the PM of SECCCs, but no candidates have been identified so far. Analysis of growth form concluded that herbaceous plants generally are not passive symplasmic loaders ^30^. Presumably, this is because active loading allows the plant to keep carbon content low in leaves, which provides a substantial, positive effect on growth rate ^31^. However, this does not mean that the slower-growing trees are all passive loaders. Indeed, half of the families dominated by woody plant species exhibit an active loading mechanism ^21, 30^. Evolutionary and distribution analyses similarly do not provide a clear explanation for why certain families use a particular phloem loading type ^30^. A closer examination of a plant’s ecology might reveal ecophysiological adaptations that different loading types offer. For example, a link between loading type and a plant’s adaptive environmental response has been demonstrated in a comparative study of minor vein structure developed under different temperature and light conditions ^32^.

### Regulation of phloem loading

While sugar export rates are a function of sugar availability and sink demand ^33, 34^ as well as phloem loading capacity ^35, 36^, the underlying molecular mechanisms are poorly understood. SUT activities are expected to be principal regulators of sugar export in apoplasmic loaders ^6^ as demonstrated by SUT overexpression ^37^. In this context, transcriptional SUT regulation by phytohormones and corresponding changes in sugar export rates were observed in potato ^38^. Several studies suggest that post-translational regulation of SUTs contributes to determining export rates (for example, Sakr *et al*. ^39^). How post-translational regulation of SUTs occurs *in vivo* is unclear but could involve differential intracellular localization ^40^, dimerization ^41^, and protein-protein interactions ^42^. However, other factors should be considered. For instance, simultaneous upregulated expression of SUTs and SWEETs, in response to water deficit, increased phloem loading ^43^. Furthermore, constitutive overexpression of a proton-pumping pyrophosphatase in *Arabidopsis thaliana* leaf CCs increased phloem loading possibly by enhancing the proton motive force driving sucrose flux through SECCC localized SUTs ^44, 45^. Regulation of sucrose exchange between cytosol and vacuole of cells in the pre-phloem pathway influences phloem loading rates even in apoplasmic loaders ^46, 47^. So far not demonstrated is the short-term regulation of phloem loading through modification of PD permeability, which would be especially relevant in symplasmic loaders ^21^. After elucidation of the molecular mechanisms regulating phloem loading, the important question will be how these are linked to sink demand ^48^.

## Phloem transport

This section combines a description of the transport phloem, especially how its anatomy facilitates sap flow, with a more general discussion on the mechanism of phloem transport and the molecules transported within.

### The mechanism of phloem transport in trees

**Figure 2.**
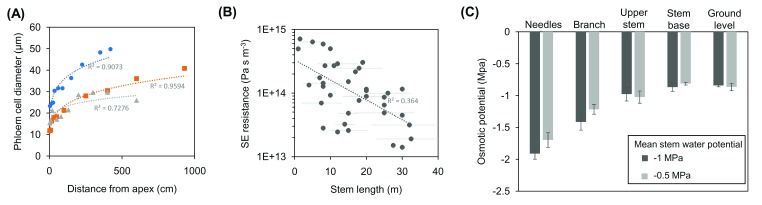
Scaling of sieve element (SE) conductivity and osmotic pressure potential enable Münch-type pressure flow in trees. ( **A**) Axial widening of phloem cells along the stem of individual trees of Norway spruce (blue), European ash (orange), and bitter willow (gray). Average values of cell diameter contain up to 30% non-SE cells. Axial widening of SEs toward the bottom of a tree translates to decreased hydraulic resistance, and the scaling relationship was found to enable optimal xylem-phloem water exchange that is critical to drive flow in tall trees. ( **B**) SE resistance of 44 different tree species with sampled individuals grown to their typical maximum height. Negative scaling of resistance with transport distance illustrates the anatomical optimization of SEs for phloem transport in trees. ( **C**) Measurements of osmolality indicated the presence of an osmotic pressure gradient along the phloem of 18 m high Scots pine trees, which changed in accordance with the xylem water potential. The differences in osmotic potential were calculated to be large enough to overcome the xylem water pressure potential and establish a phloem turgor pressure gradient that drives mass flow according to the Münch mechanism across the diel cycle ^56^. Frames ( **A**– **C**) are based on data from Petit and Crivellaro ^57^, Liesche *et al*. ^60^, and Paljakka *et al*. ^61^, respectively.

Osmotically driven pressure flow has been widely accepted as the mechanism of phloem transport in herbaceous plants ^49, 50^. However, in regard to trees, where distances between source and sink can extend up to 100 m, there are doubts about whether a hydrostatic pressure potential sufficient to drive flow could be generated ^51, 52^. A variety of approaches have been employed to answer this question. Simple theoretical models of Münch-type pressure flow agree with measurements of stem diameter variations ^53^ or SE anatomy ^54, 55^. Importantly, the main prediction of pressure flow, that SE conductivity scales with transport distance ^56^, could be confirmed. The scaling of SE conductivity with tree height was shown within a single tree ( Figure 2A; ^57, 58^), within a species ^59^, and across species ( Figure 2B; ^55, 60^), confirming that resistance decreases to accommodate mass flow in larger trees. Furthermore, it was recently shown in mature, field-grown Scots pine trees that there is an osmotic pressure gradient along the phloem pathway from leaves to the stem base ( Figure 2C; ^61^). The osmotic pressure gradient, supported by gravity, was calculated to be large enough to overcome the xylem water pressure potential and establish a phloem turgor pressure gradient that drives mass flow according to the Münch mechanism at all times across the diel cycle ^61^. Taken together, these results confirm that Münch-type pressure flow works even in the tallest angiosperm and gymnosperm trees, although transport speed might be up to 10 times lower than in herbaceous plants ^62^. However, this is in agreement with equally lower rates of photosynthesis and growth ^63^.

### Open questions regarding the functional anatomy of the phloem

Despite major progress in determining how phloem anatomy facilitates osmotically driven pressure flow, not all questions regarding its functional anatomy have been resolved. For example, sieve plates forming the axial connection between adjoining SEs have been assumed to evolve toward a simple form associated with lower hydraulic resistance—a paradigm overturned by an anatomical analysis of 447 species ^60^. Rather, the distinct organizational patterns of sieve plates might relate to general vascular anatomy (for example, SE length or xylem cell anatomy) instead of differences in conductivity ^60^. Another aspect that is relevant for phloem transport is the symplasmic coupling along the transport path. Even in apoplasmically loading herbaceous plants, unloading along the stem can switch between apoplasmic and symplasmic unloading pathways, depending upon the source/sink ratio, with high ratios favouring symplasmic unloading ^64^. In many tree species, especially conifers, the presence of PD connecting the SECCC with surrounding cells ^65^ indicates symplasmic coupling, presumably to efficiently supply the cambium with the large amounts of sugars needed for wood formation. The question is in how far symplasmic coupling along the path compromises whole-plant phloem transport. A recent theoretical analysis showed that unloading along the transport path considerably influences flow if it is not balanced by reloading ^66^. The most likely explanation is that unloading is symplasmic only in case of high sugar concentrations in the ground tissue, which then could also have a function in homeostatically maintaining SE turgor ^67^.

Another central issue is the relationship between fibrous SE protein (p-protein) agglomerations, considered to occlude sieve pores to prevent sap loss in the event of damage ^68^. Careful preparation of material for electron microscope examination reveals unobstructed SE lumens bounded by a parietal cytoplasm. However, Froelich *et al*. ^69^ showed that in *A. thaliana* phloem, the p-protein, AtSEOR1, forms a meshwork at the margins and clots in the lumen of intact SEs and their presence does not impede longitudinal flow. Together with discrepancies between theoretical flow speeds calculated according to SE conductivity and observed flow speeds ^70^, these observations have led to the conclusion that phloem transport in herbaceous plants and grasses is not limited by SE conductivity but only by sink strength ^71^.

### Phloem sap composition

Knowledge of phloem sap composition is relevant to reach a quantitative understanding of resource allocation and inter-organ signalling. Sugar concentrations are typically around 20%, but the sap contains other molecules (for example, amino nitrogen compounds, proteins, and RNAs) and mineral ions ^72^. Determining the precise abundance of the different sap components remains one of the biggest challenges in phloem research as all methods available so far are prone to artifacts ^73– 75^. Recently, laser-capture microdissection was used to uncover the link between seasonal differences in phloem metabolites and phloem formation in Norway spruce. However, since sampling could not be restricted to SEs, results were not representative of phloem sap ^76^. In the future, insight on relative sap composition might be gained from using rapid freezing and nanoscale secondary ion mass spectrometry (NanoSIMS), which enables semi-quantitative imaging of ions as well as isotope-labeled nitrogen and carbon compounds within SEs ^77^. Gaining a complete picture of the spatiotemporal dynamics of phloem sap composition will be instrumental in refining multi-compartmental metabolic models describing source-to-sink metabolite flows (for example, Zakhartsev *et al*. ^78^).

Controversy persists surrounding the function of phloem-mobile proteins and RNA ^79^. Recently, a meta-analysis comparing proteomes of xylem, phloem, and leaf apoplasmic saps highlighted phloem sap as being the most enriched in signalling proteins of which 13 were conserved across experiments and that included the ubiquitous phloem-mobile protein FLOWERING LOCUS T ^80^. Grafting studies suggest phloem mobility of a multitude of additional proteins ^81^. However, without further validation of their signalling function, many of these proteins could have entered the translocation stream “by accident” rather than by a control mechanism located at PD interconnecting CCs with SEs ^82^. Of the proteins identified in phloem sap by proteomics, those associated with redox regulation and plant defense consistently have been found across species ^80^, suggesting that many phloem proteins function in the response to biotic and abiotic stress. Prominent examples of phloem-mobile RNAs include the transcription factors *BEL5*, which influences potato tuber induction ^83^, and the Cucurbit *NACP* regulating apical meristem development ^84^. Recently 2,006 *A. thaliana* phloem-mobile mRNAs were identified ^85^ with a conserved tRNA-derived sequence conferring mobility ^86^. However, since most of the mRNAs are expressed in mesophyll cells, it is unclear how they could enter the phloem stream in the apoplasmic loader *A. thaliana*. In addition to those many phloem-mobile proteins and mRNAs, the role of microRNAs ^87^ and lipids ^88^ as systemic signals needs to be further explored.

## Phloem unloading

**Figure 3.**
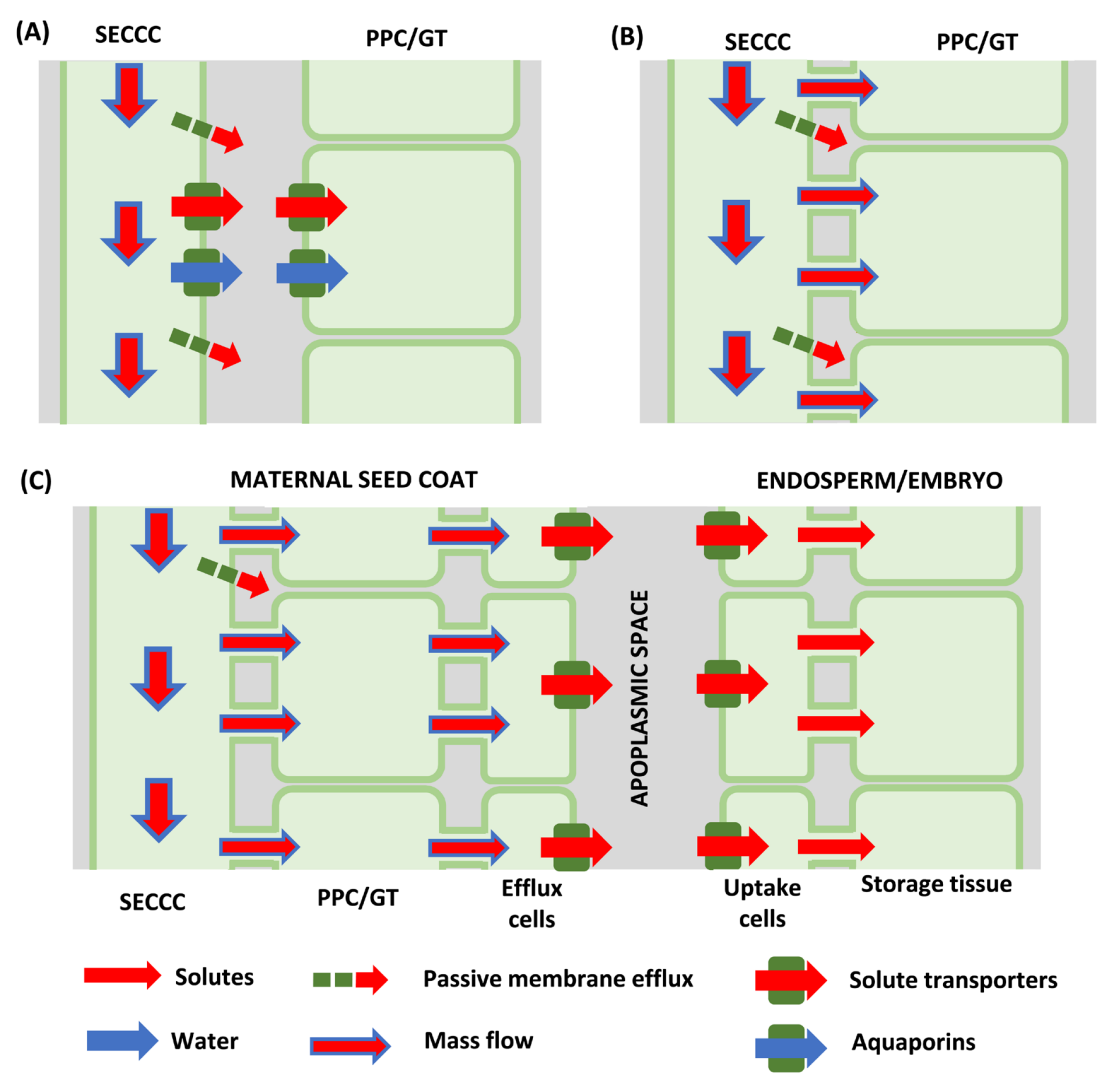
Cellular pathways and mechanisms of phloem unloading. ( **A**) Apoplasmic sieve element-companion cell complex (SECCC) unloading of sucrose mediated by sucrose transporter reversal and/or possible by sugars will eventually be exported transporters (SWEETs) with energy coupled transporters retrieving sugars from the sink apoplasm while accompanying water transport across the plasma membranes facilitated by aquaporins. ( **B**) Symplasmic phloem unloading by bulk flow. ( **C**) Symplasmic phloem unloading by bulk flow with an intervening apoplasmic step in the post-phloem pathway of developing seeds. Sucrose release to the seed apoplasm from maternal seed coats is mediated by SWEETs, sucrose facilitators and possibly a yet-to-be-cloned sucrose/proton antiporter. SWEETs and sucrose transporters recover released sucrose from the seed apoplasm into the endosperm/embryo. Membrane transport of water facilitated by aquaporins. GT, ground tissue; PPC, phloem parenchyma cell.

Phloem unloading describes the movement of phloem sap constituents from SECCC lumens (SECCC unloading) and their subsequent cell-to-cell transport to final destinations in non-SECCC vascular or ground tissues. Cellular pathways of phloem unloading are apoplasmic ( Figure 3A) or symplasmic with or without an intervening apoplasmic step located in the post-SECCC pathway ( Figure 3B, C; ^89^). As outlined below, phloem unloading mechanisms are only partially resolved.

### Apoplasmic phloem unloading mechanisms

Possible mechanisms contributing to efflux across any cell PM, including SECCC PMs, are passive diffusion, transporter-mediated facilitated diffusion, and energy-coupled movement. Passive diffusive fluxes of molecules and ions are the product of their membrane permeability coefficients and *trans*-membrane gradients in chemical potential for non-electrolytes or electrochemical potential for electrolytes ^90^. To illustrate the magnitude that these passive fluxes could reach for apoplasmic SECCC unloading of sucrose along the entire phloem pathway from source to sink, a plausible maximum of 1 M for the SECCC *trans*-PM sucrose concentration difference and a membrane permeability coefficient of 10 ^−10^ m s ^−1^ ^91^ predict a sucrose efflux of 1 × 10 ^−7^ mol m ^−2^ s ^−1^. This matches the maximal membrane flux for loading sucrose into SECCCs (2.3 × 10 ^−7^ mol m ^−2^ s ^−1^; Giaquinta ^92^). Thus, it is imperative to experimentally determine rates of diffusive leak from SECCCs and include this component in theoretical models of phloem transport.

For facilitated membrane efflux of sugars by uniporters, SWEETs ^11, 12^ have been detected in transport phloem parenchyma cells ^93^, but whether these function in efflux to, or retrieval from, the phloem apoplasm remains to be determined. However, sucrose/proton symporters occur in SECCCs of root and stem transport phloem (for example, 94, 95). Depolarized membrane potentials (−55 mV) of root SECCCs predict that sucrose/proton symport could reverse to an efflux mode driven by a *trans*-membrane sucrose concentration difference of 85.5 mM (for more information, see Carpaneto *et al*. ^94^). In contrast, symporter reversal in stem phloem is highly unlikely as their SE/CC membrane potential of −110 mV ^96^ would require a sucrose concentration difference of 7,200 mM, a concentration difference that far exceeds physiological limits.

During sugar accumulation in fleshy fruits, phloem unloading follows an apoplasmic route ^64, 97– 100^. Whether the sucrose leak from fruit SECCCs is augmented by reversal of sucrose/proton symporters ^101, 102^ cannot be evaluated, as their membrane potentials are unknown. However, pharmacological studies suggest that sucrose efflux from vascular bundles of apple fruit and grape berries is mediated by energy-coupled carriers ^97, 103^. Resolving the identity (or identities) of the putative effluxer (or effluxers) is central to acquiring a full understanding of phloem unloading in fleshy fruits. What is clearer is that monosaccharide/proton symporters retrieve inverted disaccharides from the fruit apoplasm into storage parenchyma cells ^104– 107^ and, together with cell wall invertases, function to co-regulate phloem unloading ^106, 108, 109^.

In all cases, unloading of osmotic solutes must be accompanied by a proportionate loss of phloem water to maintain water potential equilibrium. The exit of water likely occurs through aquaporins localized in PMs of the unloading SEs ( Figure 3A and, for example, 110, 111).

### Symplasmic unloading mechanisms with or without an intervening apoplasmic step located in the post-SECCC pathway

In recent years, it is becoming increasingly clear that a major component of symplasmic unloading from SECCCs is contributed by bulk flow. For instance, experimental manipulations of hydrostatic pressure gradients in root tips and stems cause changes in phloem unloading rates consistent with bulk flow (reviewed by Patrick ^112^). In developing wheat seeds, hydrostatic pressure differences of up to 1.0 MPa between SEs and vascular parenchyma cells account for observed PD volume flow rates ^113^. In contrast, modelling flows through large-diameter funnel-shaped PD, interconnecting SEs, and adjacent pericycle cells in *A. thaliana* root tips predicted that hydrostatic pressure differences of only 0.05 to 0.2 MPa were required ^114^. Whether these model-based hydrostatic pressure differences can be reconciled with measured hydrostatic or osmotic pressures of 1.3 and 0.6 MPa, respectively, between SEs and surrounding cells in barley and maize root tips ^115, 116^ awaits determination.

For the apoplasmic step in the mandatory phloem-unloading pathway of developing seeds ( Figure 3B), sucrose release from maternal seed coats occurs by a combination of facilitated diffusion and sucrose/proton antiport while retrieval by embryo/endosperm is mediated by sucrose symporters ^117^ or SWEETs ^118^ or both. Cloned sucrose facilitators (SUFs) from grain legume seed coats exhibited transport properties consistent with those found for facilitated diffusion of sucrose in native membranes of seed coats and were expressed in cells considered responsible for sucrose efflux ^119, 120^. In developing *A. thaliana* seeds, SWEETs are positioned to provide a cascade of sucrose transport from the outer (SWEET15) and inner (SWEET12) integuments and uptake into the filial tissues (SWEET11 and SWEET15 ^121^) while SWEET11 facilitates release from maternal tissues of developing rice seeds ^122^. Consistent with relative contributions of facilitated diffusion to sucrose effluxed from grain legume seed coats ^117^, seed weights, but not number, were depressed by 50% in the *A. thaliana* triple-mutant *sweet11, 12* and *15*, and by 65% in the rice *sweet11* mutant ^121, 122^. The challenge now is to identify the gene or (genes) encoding the membrane protein (or proteins) responsible for energy-coupled sucrose/proton antiport from maternal seed tissues ^123^ that may also operate in fleshy fruit (see previous section). Membrane transport of solutes is matched by water movement through aquaporins located in PMs of maternal and filial tissues of developing seeds ( Figure 3C and, for example, 124, 125).

## Conclusions and future directions

Throughout the text, we have highlighted a series of unresolved questions of phloem transport biology required to move the understanding of phloem loading, axial transport, and unloading forward. Their resolution will inform profitable approaches to address the key question of how the components of phloem transport are integrated into a functional whole ^71^ and how phloem transport mechanistically intermeshes with photosynthesis and sink demand for resources ^63, 71^.

## Abbreviations

CC, companion cell; PD, plasmodesmata; PM, plasma membrane; SE, sieve element; SECCC, sieve element-companion cell complex; SUT, sucrose transporter; SWEET, sugars will eventually be exported transporters
